# Measuring picometre-level displacements using speckle patterns produced by an integrating sphere

**DOI:** 10.1038/s41598-023-40518-6

**Published:** 2023-09-05

**Authors:** Morgan Facchin, Graham D. Bruce, Kishan Dholakia

**Affiliations:** 1 SUPA, School of Physics and Astronomy, University of St Andrews, North Haugh, St Andrews, KY16 9SS UK; 2 Department of Physics, College of Science, Yonsei University, Seoul, 03722 South Korea; 3 School of Biological Sciences, The University of Adelaide, Adelaide, SA Australia

**Keywords:** Applied optics, Optical physics

## Abstract

As the fields of optical microscopy, semiconductor technology and fundamental science increasingly aim for precision at or below the nanoscale, there is a burgeoning demand for sub-nanometric displacement and position sensing. We show that the speckle patterns produced by multiple reflections of light inside an integrating sphere provide an exceptionally sensitive probe of displacement. We use an integrating sphere split into two independent hemispheres, one of which is free to move in any given direction. The relative motion of the two hemispheres produces a change in the speckle pattern from which we can analytically infer the amplitude of the displacement. The method allows a noise floor of 5 pm/$\sqrt{\hbox {Hz}}$ ($\lambda /160,000$) above 30 Hz in a facile implementation, which we use to measure oscillations of 17 pm amplitude ($\lambda /50,000$) with a signal to noise ratio of 3.

## Introduction

The ability to measure sub-nanometre displacements is increasingly vital to a wide range of fields of science and technology. State-of-the-art gravimeters and seismometers seek to measure sub-nanometre changes in the position of oscillators^1^. As the semiconductor industry moves inexorably to smaller scales, the demands on the accurate positioning of components grows, and already requires sub-nanometre precision^2^. A related problem is found in super-resolution microscopy. While techniques such as PALM^3^ and STORM^4^ allow imaging with nanometre-resolution, the long acquisition times require the samples under investigation to drift by significantly smaller distances over the timescale of the experiments. Progress in super-resolution microscopy therefore requires simultaneous improvement of feedback-controlled sample positioning with sub-nanometre stability to avoid motion-blur.

A number of methods have emerged to resolve displacement at or below the nanometre level^5–7^. For example, tracking the light scattered by a point emitter^8^ or a nano-antenna^9^ can resolve displacements at sub-nanometre level. Phase variations of the superoscillatory fields generated by a Pancharatnam-Berry metasurface can resolve displacements of the metasurface at the nanometre-level^10^, while the transverse displacement of two such phase gratings can be detected by the polarisation rotation this encodes on a laser beam, with 0.4 nm resolution (i.e. $\lambda /1600$, where $\lambda$ is the wavelength of the light) demonstrated^11^.

Going beyond traditional one-dimensional interferometers, a powerful tool for optical metrology can be provided by the two-dimensional random interference patterns known as speckle. Such speckle patterns are produced when light interacts with a disordered medium. They typically allow for facile, inexpensive setups, and have recently been applied to various topics in metrology^12–21^.

On the particular topic of displacement, earlier works made use of speckle to measure in-plane displacement^22^ and spatially map displacement fields^23^. Many improvements and variations of these have since been made^24^. The current most accurate speckle-based method for displacement measurement, to our knowledge, tracks singularities in the pseudo phase of a moving speckle pattern to reach nanometric precision^25,26^. These methods rely on the ability to directly image the translation of features of interest, in a regime where the speckle pattern translates along the direction of displacement of the scattering medium. We suggest a fundamentally different and more sensitive approach, which is to find a geometry where small displacements induce a large morphological change in the speckle pattern. This obviates the need for any magnification system to resolve translational motion. Although speckle can be generated in many ways, it was recently analytically shown that an integrating sphere offers an optimal scattering medium, due to the large spread of optical path lengths inherent in this geometry^27^.

Here, we show that the speckle from an integrating sphere also provides a highly sensitive probe of displacement. In contrast to previous usage, we use a novel integrating sphere geometry comprising of two unattached hemispheres. The resulting speckle is recorded on a camera and shown to be extremely sensitive to the relative motion of the two hemispheres. Displacement is retrieved by evaluating the speckle similarity, that we relate to displacement following an analytical model. In contrast to previous studies measuring displacement with speckle, we implement the approach of measuring variations around the inflection point of the similarity profile, to exploit the maximum gradient of similarity. This allows us to obtain picometric sensitivity.

## Similarity profile

We first want to characterise analytically the change in the speckle pattern resulting from displacement. Consider an integrating sphere split into two independent hemispheres, one of which is able to move via a translation of amplitude *x* in any given direction. In order to infer the displacement from the speckle change, we need a quantitative tool to measure that change. We use the speckle similarity, or correlation, which is given by

1 $$\begin{aligned} S= \Big \langle \Big ( {\displaystyle \frac{\displaystyle I - \langle I\rangle }{\displaystyle \sigma _{I}}} \Big )\Big ( \frac{I'-\langle I'\rangle }{\sigma _{I'}} \Big ) \Big \rangle , \end{aligned}$$

with *I* and $I'$ two speckle images, $\sigma _{I}$ and $\sigma _{I'}$ their respective standard deviation, and the angular brackets denote averaging over the image. This gives a value of 1 for identical images, and decreasing values as they diverge from one another.

In the case of speckle patterns produced by multiple reflections of light in an integrating sphere, it was shown in Ref. ^27^ that for two speckle patterns taken before and after an arbitrary transformation, the similarity is given by

2 $$\begin{aligned} S = \frac{ 1 }{\left( 1-{\displaystyle \frac{\displaystyle \sigma ^2}{\displaystyle 2\ln {\rho }}} \right) ^2+\left( {\displaystyle \frac{\displaystyle \mu }{\displaystyle \ln {\rho }}} \right) ^2 }, \end{aligned}$$

where $\mu$ and $\sigma ^2$ are respectively the average and variance of the phase shift induced by the transformation on a single pass of light through the sphere, with $\rho$ the sphere’s surface reflectivity. A single pass (or chord) is defined as a straight line joining two points of the sphere. This expression assumes that the inner surface of the sphere has a Lambertian reflectance with uniform reflectivity.

**Figure 1:**
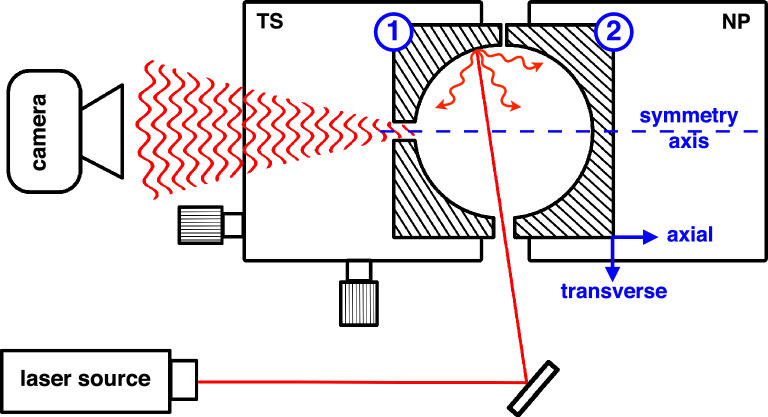
Experimental setup. Laser light enters the integrating sphere and produces a speckle pattern recorded on a camera. Hemisphere (1) rests atop a manual 3D translation stage (TS) for coarse alignment, hemisphere (2) rests atop a motorised nanopositioner (NP). The latter is moved at a constant speed while the changing speckle pattern is recorded. The symmetry axis, as well as the axial and transverse directions are shown.

In our case, the phase shift is related to the change in length of the chords resulting from the displacement. We derive analytically the expressions of $\mu$ and $\sigma ^2$ (see Supplementary Material) in two particular cases, when the displacement is along the symmetry axis (denoted as axial motion), and perpendicular to the symmetry axis (denoted as transverse motion). The symmetry axis and both directions are shown in Fig. 1.

In the axial case, we find $\mu =kx/3$ and $\sigma =kx\sqrt{5}/6$ (eqs. (S6) and (S7)), with *k* the wavenumber, and *x* the amplitude of the displacement. Inserting this in (2), we can neglect the $\sigma$ term and obtain

3 $$\begin{aligned} S= {\displaystyle \frac{\displaystyle 1}{\displaystyle 1+\left( \frac{kx}{ 3\ln {\rho }}\right) ^2 }}, \end{aligned}$$

which is a Lorentzian with an HWHM (half width at half maximum) of $3\lambda \left| \ln {\rho }\right| /2\pi \approx 0.5\lambda \left| \ln {\rho }\right|$.

In the transverse case, we have $\mu =0$ and $\sigma =kx/\sqrt{8}$ (eqs. (S8) and (S9)), leading to

4 $$\begin{aligned} S= {\displaystyle \frac{\displaystyle 1}{\displaystyle \left( 1-\frac{\left( kx\right) ^2}{16\ln {\rho }} \right) ^2 }}, \end{aligned}$$

which is the square of a Lorentzian, with an HWHM of $\sqrt{16(\sqrt{2}-1)}\lambda \sqrt{\left| \ln {\rho }\right| }/2\pi \approx 0.4\lambda \sqrt{\left| \ln {\rho }\right| }$.

The axial motion imparts a greater change to the speckle pattern than the transverse one. This can be understood qualitatively, as in the axial case the chords increase in length on average, resulting in a systematic phase increase after each single pass. In the transverse case however, some chords can compensate each other, leading to smaller change.

For typical parameters such as $\lambda =780$ nm and $\rho =0.9$, the HWHM is 39 nm in the axial case, and 104 nm in the transverse case. We note that the HWHM is not the resolution limit of the approach - the ultimate resolution of the method depends on the smallest detectable variation in similarity. Note also that the sensitivity of the speckle pattern to displacement is independent of the size of the sphere, contrary to what is found for wavelength and refractive index variation^27^.

The knowledge of the similarity profiles allows one to infer the displacement between two given instants, by simply applying the reciprocal function of the appropriate profile and using the similarity between the two corresponding speckles. For example, for the axial profile, using (3) we have:

5 $$\begin{aligned} x = x_0 \sqrt{1/S-1}, \end{aligned}$$

with $x_0$ the HWHM.

## Experimental implementation

**Figure 2:**
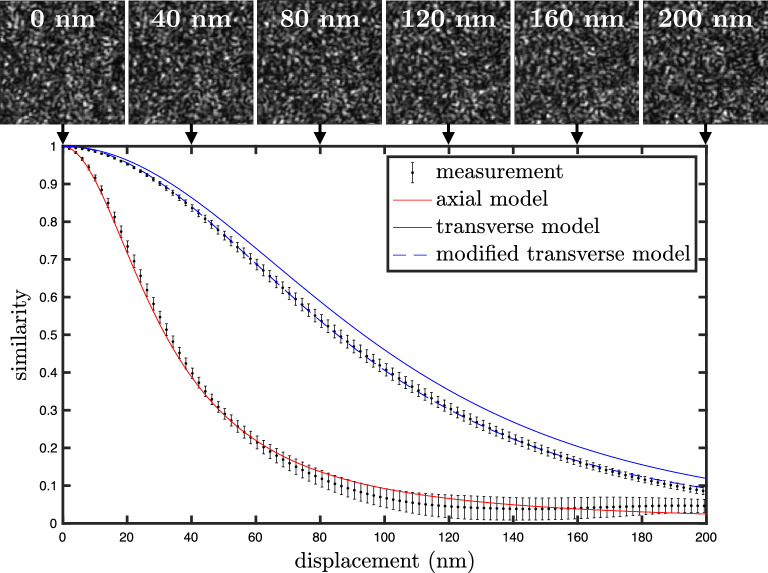
Speckle similarity profiles as a function of displacement. Experimental data (black), axial theoretical profile (red), and transverse theoretical profile (blue). The HWHM is 32 nm in the axial case and 85 nm in the transverse case. An image of the changing speckle is shown on top of the graph at regular spacing in the case of transverse displacement. The observed transverse profile shows a small systematic deviation from the model, which can be explained by an imperfect Lambertian reflectance and/or a non uniform reflectivity. This is taken into account in the modified model (dashed blue).

In this section we experimentally verify the relations (3) and (4) with the setup described in Fig. 1. A laser beam of 780 nm wavelength, 20 mW power, and a coherence length of a few kilometres (Toptica DLPro) is injected into an integrating sphere, and the resulting speckle pattern is collected on a CMOS camera (Mikrotron MotionBLITZ EoSens mini2), located 20 cm away from the integrating sphere. This distance determines the size of the speckle grains, covering an area equivalent to about 100 pixels in our case. This parameter is not essential, speckle grain size can take any value as long as it is larger than a few pixels, to avoid any possible effect related to spatial averaging of the speckle within each pixel (which reduces speckle contrast, see section 4.6 of Ref.^28^). We use $200\! \times \! 200$-pixels images which offers a good compromise between computation time and variability of the computed similarity profile. We use a 1.25 cm radius sphere, carved in a 3 cm edge aluminium cube and coated with Spectraflect to give a near Lambertian reflectance with reflectivity $\rho =0.918\pm 0.008$. Light enters and exits through two 3 mm diameter holes. The sphere is made of a fixed and a moving half. The fixed half is attached to a manual translation stage, for alignment of the two parts, and the moving half is attached to a 3D precision stage capable of nanometre precision (PI P-733.3DD). We displace the moving hemisphere in the axial and transverse (horizontal) direction at a constant speed of 0.1 ${\mathrm{\mu}}{\textrm{m\,s}}^{-1}$ while the changing speckle is recorded, and extract the similarity profiles by applying expression (1) between one reference and subsequent images. The resulting profiles are shown in Fig. 2. We find a good agreement for the axial profile, while the transverse profile shows a small deviation. This difference was found to be systematic and independent of many experimental parameters. In Supplementary Material, we show how this can be explained by a small deviation from the assumptions of our model, in particular the Lambertian reflectance and the uniform reflectivity across the inner surface of the integrating sphere. By choosing different reference images, we extract different similarity profiles, which allows us to find the average and standard deviation of the profile that we display as an error bar in Fig. 2. It is worth noting that these error bars do not correspond to a measurement uncertainty, but a physical variability of the profile. When different reference points are chosen along the *x* axis, the similarity profile is not strictly identical, due to the intrinsic randomness of the system. Importantly, this variability is *not* related to the uncertainty with which an individual value of the similarity is computed between two given images. In a displacement measurement, it is this latter uncertainty that ultimately determines resolution. In Fig. 2 we also show the theoretical profiles (3) and (4), which can be determined knowing only the wavelength and the sphere’s reflectivity.

Note that the coherence length of the laser does not need to be as high as that used in this study. The standard deviation of the path length distribution of the sphere is $4R/(3 \left| \ln {\rho }\right| )$ (derived from the path length distribution given in Ref.^29^), which is equal to 19.5 cm with our parameters. The coherence length only needs to be large compared to the standard deviation of path length, therefore more standard laser sources with coherence lengths of the order of meters should be suitable.

## Variation with a virtual hemisphere

**Figure 3:**
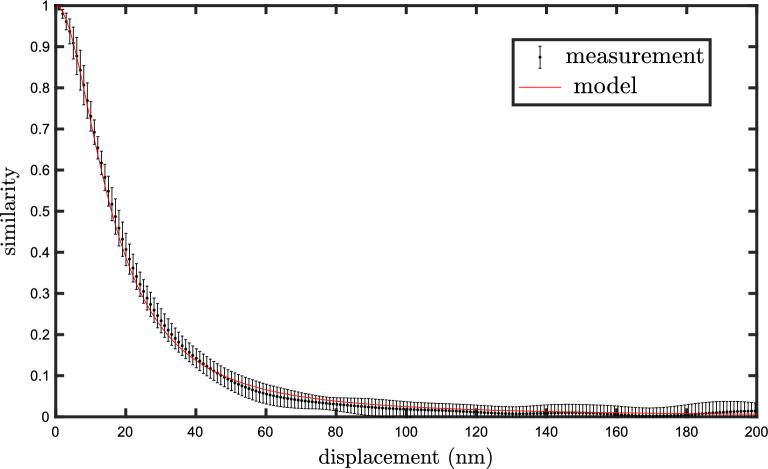
Axial similarity profile in a modified version of the experimental setup, where the moving hemisphere is replaced by a flat mirror. The profile is also Lorentzian, with a HWHM of 16 nm, precisely half that found in the previous experiment. This finds a simple explanation in terms of the moving virtual image of the hemisphere. Here the axial model is obtained by multiplying *x* by 2 in Eq. (3).

To allow the speckle to become more sensitive to axial motion and insensitive to transverse motion, we apply a small modification to the previous experimental setup, where the moving hemisphere is replaced by a flat mirror (Thorlabs BB1-E03) with reflectivity $>0.99$, orthogonal to the symmetry axis. The similarity profile obtained for the axial motion in this case is shown in Fig. 3.

Although the geometry of the problem seems very different from the previous experiment, we find again a Lorentzian profile, with a HWHM precisely twice smaller (the same factor of two was found with other spheres of different reflectivities and radii). This curious result can be understood in a simple way if we think of the image of the fixed hemisphere in the mirror (which we refer to as the virtual hemisphere). The real and the virtual hemispheres form together a complete sphere, in which we can apply our previous model as if the whole sphere was real. Indeed, the motion of the mirror implies an axial motion of the virtual hemisphere, just like we had in the previous setup. The virtual hemisphere however, by its very nature, moves twice as much as the mirror, which explains the factor of two. Also, transverse motion does not change the position of the virtual hemisphere, which is consistent with the absence of speckle change in that case.

In this interpretation we neglect the effect of the mirror’s own reflectivity (>0.99 measured at 6° and 45° angle of incidence), as it is much higher than that of the sphere (0.918). If both reflectivities were closer together, the result would be less trivial. In fact, as any path reaching the virtual hemisphere has to undergo a reflection on the mirror, the similarity profile would be that of a real sphere divided in two regions of different reflectivities, one $\rho$ and one $\rho '\rho$ (with $\rho '$ the mirror’s reflectivity), which would surely result in a similarity profile implying a non-trivial combination of $\rho$ and $\rho '$.

## Noise floor

In this section we perform the measurement of small displacements and determine the noise floor. For displacements much smaller than $x_0$, using directly (5) is not ideal, as the similarity has a derivative of zero at $x=0$. This problem would disappear if we could instead measure variations of the similarity around a point of higher slope along the profile, and most optimally at the inflection point where the slope is maximal. This can be done by purposely applying an initial displacement, prior to any measurement, that takes the similarity to its inflection point. Instead of applying an initial displacement however, we apply a wavelength variation instead, which has the same physical effect and allows us to control the initial offset independently. Indeed, any transformations that are dominated by the $\mu$ term in (2) can be used interchangeably to produce the same change in the final speckle pattern (thermal expansion and refractive index variation are other examples^20^). The inflection point of the axial profile takes place at $x=x_0/\sqrt{3}$, which corresponds to $S=3/4$. The wavelength variation is applied by varying the diode current of the laser, and chosen so that the similarity drops to a value close to 3/4 (the order of magnitude is $\sim \Delta \lambda =0.5$ pm). The speckle before and after the variation are recorded, the similarity between them is computed, that we shall denote $S_\lambda$, and the equivalent displacement is $x_\lambda =x_0\sqrt{1/S_\lambda -1}$. Subsequent measurements of *S* correspond to a displacement $x=x_0\sqrt{1/S-1}=x_\lambda +\delta x$, with $\delta x$ any subsequent displacement. It follows that $\delta x$ is given by

6 $$\begin{aligned} \delta x=x_0\sqrt{1/S-1}-x_\lambda . \end{aligned}$$

Note that the precise value of the initial wavelength shift need not be known, as it does not come into play at any stage of the calculations. We only need the similarity to be brought close to 3/4. Also, in computing (6), only one reference image is used, and $\delta x$ is the displacement relative to the time of that reference image.

**Figure 4:**
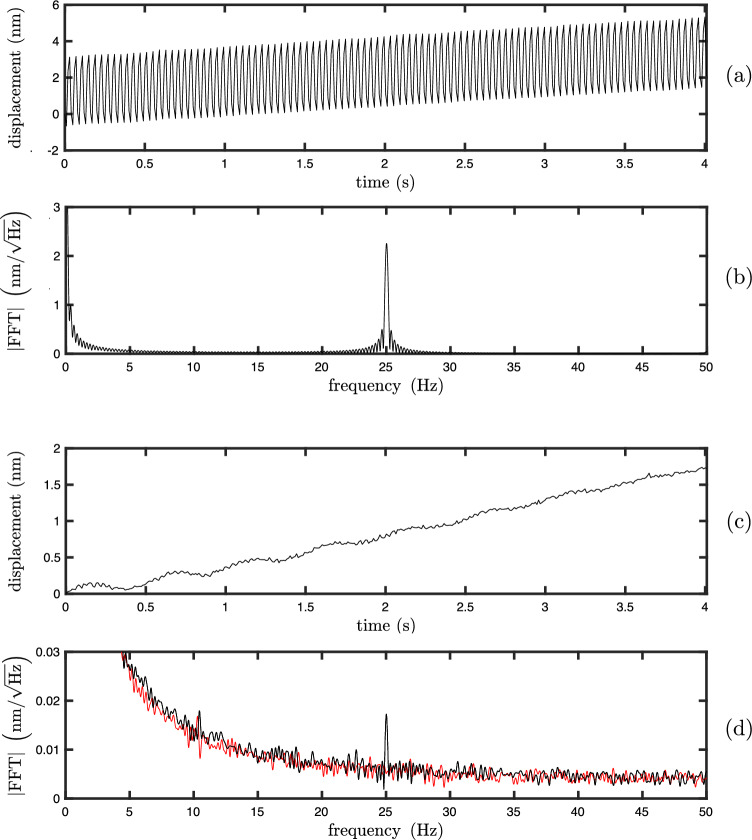
(**a**) and (**b**): measured displacement as a function of time and its Fourier spectrum when a 25 Hz modulation of 2.3 nm amplitude is applied. (**c**) and (**d**): measured displacement as a function of time and its Fourier spectrum when a 25 Hz modulation of 17 pm amplitude is applied. In panel (**d**) is also shown the Fourier spectrum of displacement when no modulation is applied (red). In each case, we record 4 s at a sampling frequency of 100 Hz.

We apply this approach in the hemisphere-mirror configuration described in the previous section, so that only the axial component is probed. A gap of $\sim$0.5 mm is left between the hemisphere and the mirror, and the mirror is attached to a piezoelectric crystal (APC 70-2221) to apply controlled sinusoidal displacements. For a 25 Hz modulation with 2.3 nm amplitude, the displacement is found using (6) and shown in Fig. 4a, and its Fourier spectrum in Fig. 4b. The linear increase in displacement is due to the thermal expansion of the sphere resulting from the input laser power, which is also responsible for the low frequency peak in the Fourier spectrum. We then decrease the amplitude of the modulation until it becomes discernable with a signal to noise ratio of about 3. We reach a modulation amplitude of 17 pm, the measured displacement is shown in Fig. 4c and its Fourier spectrum in Fig. 4d. We also show in red in Fig. 4d the Fourier spectrum of displacement when no modulation is applied, which gives the fundamental noise level of the measurement. This is below 14 pm/$\sqrt{\textrm{Hz}}$ above 10 Hz, and reaches a plateau of 5 pm/$\sqrt{\textrm{Hz}}$ above 30 Hz. In each case of Fig. 4, we record 4 s at a sampling frequency of 100 Hz, and the Fourier spectrum is found by taking the absolute square of the Fourier transform of x(t).

## Sources of error

The main source of error is camera noise on each individual pixel that propagates to the value of the similarity. As we work in conditions of high illumination and short exposure times, the effect of photon shot noise dominates the other sources of noise (such as dark current, readout, and quantisation). By nature of the photon shot noise, the determinant factor is the total number of photons detected. This depends primarily on the total number of pixels in the image, and marginally on other camera parameters such as quantum efficiency and filling factor.

While the measurement uncertainty is much lower than $x_0$, the fractional uncertainty of $x_0$ still contributes to a source of error. As $x_0$ appears as a global factor in the computation of the displacement (5) and (6), the fractional uncertainty on displacement will be the same as the fractional uncertainty on $x_0$. As the same value of $x_0$ is used for all measurements, this translates into a systematic error. With the value of the HWHM of the axial profile given above ($x_0=32\pm 3$ nm), this corresponds to a fractional uncertainty of 9$\%$.

The heating effect, responsible for the linear trend in the measured displacement in Fig. 4, can be mitigated by either applying a real time compensation^20^, by using Fourier filtering, or by using the device as a velocimeter or accelerometer instead. In a more engineering perspective, one could also choose the route of preventing the heating itself via temperature stabilisation of the sphere. Fourier filtering is a particularly convenient route, as the effect of heating and the signal of interest can be easily distinguished in the Fourier domain (if the frequency content of the signal is high enough).

The performance of the method can be improved mainly via three parameters: the wavelength, the sphere’s reflectivity, and the number of pixels in the image. On one hand, the smallest detectable displacement scales with the width of the similarity profile, which depends directly on the wavelength and the sphere’s reflectivity, as shown by Eqs. (3) and (4). Cone, et al, have demonstrated diffusely reflecting surfaces with reflectivity up to 0.99919 at 532 nm^30^. With these parameters, the combined effect of the higher reflectivity and shorter wavelength would give an axial HWHM of 206 pm (or 155 times smaller). Finally, the effect of photon shot noise can be decreased by increasing the number of pixels.

The measurement of large displacements, by direct application of (5), is limited to a size comparable to the HWHM. This implies that the dynamic range of the instrument, defined as the ratio between the largest and smallest measurable displacement (32 nm and 27 pm respectively), is of the order of 2000. The measurement of displacements beyond the HWHM could be made possible by keeping track of the speckle variation over time, analogously to what is done with standard interferometers.

## Discussion

In this work, we studied analytically and experimentally the sensitivity of the speckle patterns to the relative displacement of two hemispheres forming an integrating sphere. We demonstrated that, by replacing one hemisphere with a highly-reflective mirror, the displacement sensitivity is enhanced by a factor two in the axial direction while vanishing in the transverse direction, which gives an elegant method to selectively probe one direction of motion. Using this hemisphere-mirror configuration, we showed that the noise floor is 5 pm/$\sqrt{\textrm{Hz}}$ above 30 Hz, and that a sinusoidal displacement of 17 pm amplitude (or 6 times smaller than a hydrogen atom) could be resolved with a signal to noise ratio of 3.

The method presented here has the advantage of a very simple implementation, as it only involves a single beam with no special alignment needed, with the two hemispheres only requiring coarse alignment. Moreover, as the displacement is captured by a morphological change in the speckle as opposed to a small translation of a feature of interest, the approach does not require expensive magnification systems, which is in contrast to previous approaches^10,25^.

We note that, interestingly, sensitivity is independent of the radius of the sphere, indicating that similar performance could be achieved in a more compact fashion. However the model used in this work would remain valid only for a sphere radius large enough compared to the wavelength and its surface roughness. This method could be applied to any fields where the measurement of nanometric to sub-nanometric displacements is important, such as force sensing or mechanical characterisation.

### Supplementary Information

Supplementary Information.

## Data Availability

The datasets used and/or analysed during the current study are available at 10.17630/e7798c1b-e851-449c-b360-857f70300e78.
