# Supplementary material for: Measuring picometre-level displacements using speckle patterns produced by an integrating sphere
Source: Sci Rep. 2023 Sep 5;13:14607. doi: 10.1038/s41598-023-40518-6 (PMC10480476; doi:10.1038/s41598-023-40518-6)
Supplement: Supplementary file 1 — Supplementary Information. [file 41598_2023_40518_MOESM1_ESM.pdf]

Supplementary material to "Measuring picometre-level displacements using speckle patterns produced by an integrating sphere"

Morgan Facchin[1,*], Graham D. Bruce[1,], and Kishan Dholakia[1,2,3]**

[1]SUPA, School of Physics and Astronomy, University of St Andrews, North Haugh, St Andrews KY16 9SS, UK
[2]Department of Physics, College of Science, Yonsei University, Seoul 03722, South Korea
[3]School of Biological Sciences, The University of Adelaide, Adelaide, South Australia, Australia
[*]mf225@st-andrews.ac.uk
[**]gdb2@st-andrews.ac.uk

ABSTRACT

Here we derive the axial and transverse similarity profiles, and the modified transverse model, discussed in the main text "Measuring picometre-level displacements using speckle patterns produced by an integrating sphere".

1 Derivation of the Similarity Profiles

We derive here the expression of the similarity profiles for the axial and transverse motion. We recall that the general expression of the similarity for an arbitrary transformation is

$$S = \frac{1}{\left(1 - \frac{\sigma^2}{2\ln\rho}\right)^2 + \left(\frac{\mu}{\ln\rho}\right)^2}, \tag{S1}$$

where μ and σ^2 are respectively the average and variance of the phase shift induced by the transformation on a chord through the sphere, with ρ the sphere's surface reflectivity. A chord is defined as a straight line joining two points of the sphere.

To find μ and σ^2, we first need to express the phase shift on a chord resulting from the displacement of the hemisphere. The effect of the displacement is to change the lengths of the chords, and therefore the length of propagation of light, which is why the effect takes the form of a phase shift. Along a chord of length z, light acquires a phase kz, with k the wavenumber. When the hemisphere is displaced, this phase varies by $k\Delta z$, with Δz the change in length. For small displacements, Δz is given by $u \cdot x$, with u a unit vector parallel to the chord (oriented from the fixed to the moving end), and x the displacement vector of the moving hemisphere. Moreover, no phase shift occurs if the chord starts and ends on the same hemisphere. This can be modelled by a variable s, that takes a value of 0 when both ends of the chord belong to the same hemisphere, and 1 otherwise. It follows that the phase shift induced by the displacement on a chord can be expressed as

$$\phi = k\, u \cdot x\, s. \tag{S2}$$

The terms μ and σ^2 in (S1) are the mean and variance of ϕ. We therefore seek to express the following quantities

$$\mu = \overline{\phi} \qquad \sigma^2 = \overline{\phi^2} - \overline{\phi}^2, \tag{S3}$$

where the bar indicates averaging over random chords in the sphere. A random chord is a chord whose endpoints are chosen with a uniform probability distribution across the inner surface.

Let us express ϕ in more explicit terms before computing the averages. We express u and x in a spherical coordinate system, whose origin is at the centre of the sphere and z axis confounded with the symmetry axis, with the moving hemisphere being on the positive side. In this system we use the spherical angles $\theta \in [0\ \pi/2]$ and $\varphi \in [0\ 2\pi]$. The restricted range of θ is chosen to guarantee that u is uniquely defined for a given chord, and points from the fixed to the moving hemisphere. In the axial case, we have $x = x\hat{z}$, and therefore $\phi = kx\cos\theta\, s$, with θ the angle between the chord and the symmetry axis. With this we can express μ as

$$\mu = \iint kx\cos\theta\, s\, f(\theta, s)\,\mathrm{d}\theta\,\mathrm{d}s, \tag{S4}$$

with $f(\theta,s)$ the joint probability distribution of θ and s. As s is a discrete variable, this can be recast as

$$\mu = \int_0^{\pi/2} kx\cos\theta\, s\, P(s{=}1|\theta) f(\theta)\mathrm{d}\theta, \tag{S5}$$

with $P(s{=}1|\theta)$ the probability of s being 1 for a given θ, and $f(\theta)$ the probability distribution of θ.

We can find $f(\theta)$ in the following way. By symmetry, the distribution of random chords is isotropic. Therefore, the set of all possible u vectors forms a uniform unit hemisphere. The number of chords contained around a certain θ is then proportional to the surface element in our spherical system, which is proportional to $\sin\theta$. After normalisation, we simply have $f(\theta) = \sin\theta$.

Finding $P(s{=}1|\theta)$ is more subtle. Let us consider the set of chords contained in an infinitesimal solid angle around the direction u forming an angle θ with the symmetry axis. It can be shown that those chords cross any plane perpendicular to u with a uniform density. As $P(s{=}1|\theta)$ corresponds to the proportion of those chords that touch both hemispheres, we find by geometry $P(s{=}1|\theta) = \cos\theta$ (see Fig. 1).

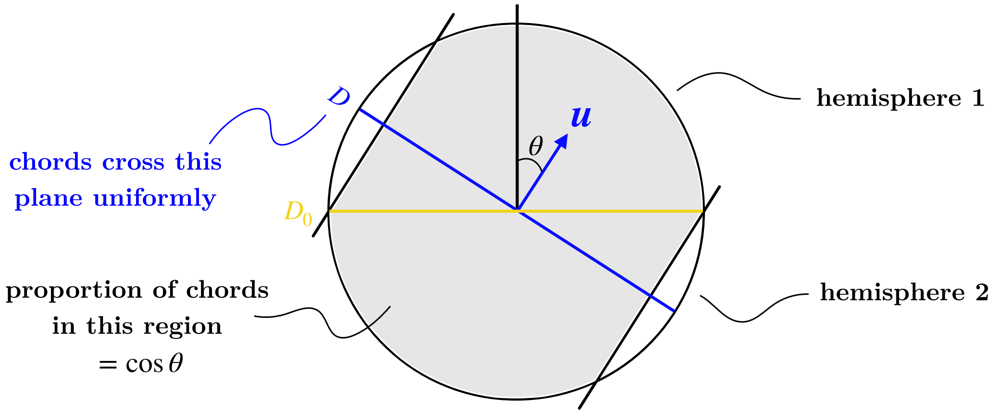

Figure 1. Side view of the sphere with the separation between the two hemispheres forming the disk D_0. The chords pointing around the direction u cross uniformly the disk D. Among those, the chords that touch both hemispheres are contained in the shaded region, that is, the "shadow" or D_0 in the direction of u. The proportion of chords in this region is equal to $\cos\theta$.

We have now everything in hand to to compute the integrals:

$$\begin{aligned}\mu &= \int_0^{\pi/2} kx\cos\theta\cos\theta\sin\theta\mathrm{d}\theta \\ \mu &= \frac{kx}{3}.\end{aligned} \tag{S6}$$

Similarly for σ:

$$\begin{aligned}\sigma^2 &= \int_0^{\pi/2} (kx\cos\theta)^2\cos\theta\sin\theta\mathrm{d}\theta - \mu^2 \\ \sigma^2 &= \frac{(kx)^2}{4} - \frac{(kx)^2}{9} = \frac{5}{36}(kx)^2.\end{aligned} \tag{S7}$$

In the transverse case, we have $x = x\hat{x}$ (or any direction in the xy plane), and therefore $\phi = kx\sin\theta\cos\varphi\, s$, with θ the angle between the chord and the symmetry axis, and φ the azimuthal angle of the chord with respect to the $\hat{x}$ direction. This invokes the distribution of the azimuthal angle $f(\varphi)$, which by symmetry is uniform, giving $f(\varphi) = 1/2\pi$. This leads to

$$\begin{aligned}\mu &= \int_0^{2\pi}\int_0^{\pi/2} kx\sin\theta\cos\varphi\cos\theta\sin\theta\frac{1}{2\pi}\mathrm{d}\theta\mathrm{d}\varphi \\ \mu &= 0\end{aligned} \tag{S8}$$

$$\begin{aligned}\sigma^2 &= \int_0^{2\pi}\int_0^{\pi/2} (kx\sin\theta\cos\varphi)^2\cos\theta\sin\theta\frac{1}{2\pi}\mathrm{d}\theta\mathrm{d}\varphi \\ \sigma^2 &= \frac{(kx)^2}{8}.\end{aligned} \tag{S9}$$

We can also perform the calculation for an arbitrary direction of displacement forming an angle β with the symmetry axis, in which case we find

$$\begin{aligned}\mu &= \frac{kx}{3}\cos^2\beta \\ \sigma^2 &= (kx)^2\left(\frac{1}{8}\sin^2\beta + \frac{5}{36}\cos^2\beta\right),\end{aligned} \tag{S10}$$

yielding the axial and transverse results from $\beta = 0$ and $\beta = \pi/2$ respectively.

Results (S6) to (S9) were verified numerically by generating a set of random points uniformly distributed across the unit sphere. The points belonging to one hemisphere were translated in either the axial or transverse direction, and the statistics of the chord length variations were computed. We found very good agreement to 1 part in 1000.

Finally, we find the axial and transverse profile by inserting the expressions of μ and σ^2 in (S1), given by (3) and (4) in the main text.

2 Modified transverse Model

In the main text, we showed that a small but significant deviation was found between the theoretical and the observed transverse profile (Fig. 2). Here we show that this can be explained by a deviation from some of the assumptions of the model, in particular the Lambertian reflectance and uniform reflectivity. When such deviations are introduced in the model, we find that it does not change the prediction of the axial profile in first approximation, but does change that of the transverse profile. The obtained modified model can be empirically adjusted to reproduce the observed profile.

Following the derivation given in ref. 26 of the main text, the general expression of the similarity (S1) comes from the integral

$$S = \left|\int_0^\infty -\ln\rho\ \left(\rho\overline{e^{i\phi}}\right)^N dN\right|^2, \tag{S11}$$

where ϕ, the phase shift applied to the light on a chord, is Gaussian with mean μ and variance σ^2, and the overline designates averaging over random chords. The term $\rho\overline{e^{i\phi}}$ in turn comes from the fact that the intensity of a light ray always decreases by the same factor after each reflection. For this reason, only the phase term is in the chord-averaging and the reflectivity ρ is outside.

We can describe any deviation from the Lambertian reflectance or the uniform reflectivity by a dimensionless function g, which we include as a factor of T. This function can contain a direction dependence to model an excess of power in the specular direction for example, or a position dependence to model a non uniform reflectivity. We have $g = 1$ for a Lambertian reflectance and uniform reflectivity, and $\overline{g} = 1$ in any case, by conservation of power. With this definition, the previous $\rho\overline{e^{i\phi}}$ term becomes $\rho\overline{ge^{i\phi}}$. The actual expression of this term would be difficult to derive, but we can still infer the effect of g on the final similarity profile. We start by expressing $\overline{ge^{i\phi}}$ as differing from $\overline{e^{i\phi}}$ by a complex number ae^{ib}, reading

$$\overline{ge^{i\phi}} = ae^{ib}\overline{e^{i\phi}}, \tag{S12}$$

with a and b unknown dimensionless functions of kx. We can be more specific on the behaviour of a and b. First, For no displacement ($x = 0$), we have $a = 1$ and $b = 0$, as $\overline{g} = 1$. Also, as kx is small in our range of measurement (0.26 for $x =$ HWHM), we can Taylor expand a and b around zero. By keeping only the first non zero terms of the Taylor expansions, we have $a = 1 + \alpha(kx)^2/2$ and $b = \beta kx$, with α and β small dimensionless numbers. Indeed, it can be shown that the derivative of a is zero at $kx = 0$ (by expanding the derivative with respect to x of the expression $\overline{ge^{i\phi}} = ae^{ib}e^{i\mu-\sigma^2/2}$). Using these expansions, we can insert (S12) in place of $\overline{e^{i\phi}}$ in (S11), perform the integral and see the impact this has on the final form. We find the modified profile

$$S = \frac{1}{\left(1 - \frac{\sigma^2 + \alpha(kx)^2}{2\ln\rho}\right)^2 + \left(\frac{\mu + \beta kx}{\ln\rho}\right)^2}. \tag{S13}$$

In the axial case, we see that the modification is negligible, as the sigma term was already negligible in the original profile, and $\mu \gg \beta kx$. In the transverse case, we have

$$S = \frac{1}{\left(1 - (1+8\alpha)\frac{(kx)^2}{16\ln\rho}\right)^2 + \left(\frac{\beta kx}{\ln\rho}\right)^2}, \tag{S14}$$

where we see that the modification is not negligible. This modified profile with two free parameters α and β gives an excellent fit of the data for $\alpha = 0.027$ and $\beta = 0$, which is an argument in favour of the hypothesis that the observed deviation does come from an imperfect Lambertian reflectance and/or a non uniform reflectivity. The modified profile is shown in Fig. 2.
